# Indole-3-acetic acid is a physiological inhibitor of TORC1 in yeast

**DOI:** 10.1371/journal.pgen.1009414

**Published:** 2021-03-09

**Authors:** Raffaele Nicastro, Serena Raucci, Agnès H. Michel, Michael Stumpe, Guillermo Miguel Garcia Osuna, Malika Jaquenoud, Benoît Kornmann, Claudio De Virgilio

**Affiliations:** 1 Department of Biology, University of Fribourg, Fribourg, Switzerland; 2 Department of Biochemistry, University of Oxford, Oxford, United Kingdom; University of Georgia, UNITED STATES

## Abstract

Indole-3-acetic acid (IAA) is the most common, naturally occurring phytohormone that regulates cell division, differentiation, and senescence in plants. The capacity to synthesize IAA is also widespread among plant-associated bacterial and fungal species, which may use IAA as an effector molecule to define their relationships with plants or to coordinate their physiological behavior through cell-cell communication. Fungi, including many species that do not entertain a plant-associated life style, are also able to synthesize IAA, but the physiological role of IAA in these fungi has largely remained enigmatic. Interestingly, in this context, growth of the budding yeast *Saccharomyces cerevisiae* is sensitive to extracellular IAA. Here, we use a combination of various genetic approaches including chemical-genetic profiling, SAturated Transposon Analysis in Yeast (SATAY), and genetic epistasis analyses to identify the mode-of-action by which IAA inhibits growth in yeast. Surprisingly, these analyses pinpointed the target of rapamycin complex 1 (TORC1), a central regulator of eukaryotic cell growth, as the major growth-limiting target of IAA. Our biochemical analyses further demonstrate that IAA inhibits TORC1 both *in vivo* and *in vitro*. Intriguingly, we also show that yeast cells are able to synthesize IAA and specifically accumulate IAA upon entry into stationary phase. Our data therefore suggest that IAA contributes to proper entry of yeast cells into a quiescent state by acting as a metabolic inhibitor of TORC1.

## Author summary

Auxins are a major group of plant phytohormones that are critical for growth and development. Amongst the auxins, indole-3-acetic acid (IAA) is the most common, naturally occurring phytohormone that regulates cell division, differentiation, and senescence in plants. Interestingly, the capacity to synthesize and secrete IAA is also widespread among fungi, including the budding yeast *Saccharomyces cerevisiae*, but the role of IAA in fungi has largely remained unknown. Here, we confirm an earlier observation that IAA inhibits growth of budding yeast and show by diverse genetic and biochemical means that IAA restrains budding yeast growth by inhibiting the target of rapamycin complex 1 (TORC1), a highly conserved eukaryotic regulator of growth. Intriguingly, budding yeast cells accumulate IAA specifically when limited for nutrients, which suggests that IAA plays a hitherto unknown physiological role in contributing to the establishment of cellular quiescence by acting as a metabolic inhibitor of TORC1.

## Introduction

Auxins are a major group of plant phytohormones that are critical for growth and development. Amongst the auxins, indole-3-acetic acid (IAA) is the most common, naturally occurring phytohormone, and is best understood for its role in regulating cell division, differentiation, organogenesis, and senescence in plants [1, 2]. Interestingly, the capacity to synthesize (and secrete) IAA is also widespread among numerous plant-associated bacterial and fungal species, and critically defines their relationship with the respective plants, which range from pathogenic to symbiotic [3, 4]. While the role of IAA as an effector molecule in these microbe-plant interactions has attracted a lot of attention, several studies indicate that IAA may also serve as a signaling molecule that coordinates the physiological behavior of and/or mediates cell-cell communication in bacteria [4, 5]. Fungi, including many species that do not entertain an apparent plant-associated life style, are also able to synthesize IAA [6, 7]. However, the role of IAA as an intracellular fungal metabolite remains unknown, and only a handful of studies have so far analyzed the effects of extracellular IAA on fungal physiology. For instance, IAA has been reported to affect the sporulation efficiency, spore germination, and filamentous growth in various fungal species [8–10]. IAA has also been found to control the hyphal transition, a key virulence trait, of the human pathogen *Candida albicans* [11]. Notably, while stimulating hyphal growth at lower concentrations (in the 2-digit micromolar range), higher IAA levels (in the millimolar range) appear to strongly reduce overall growth in diverse fungi [12–14]. Based on the currently rather circumstantial knowledge on the potential role of IAA in fungi, it has been speculated that IAA may, analogously to other compounds in bacteria [15], play a role as quorum-sensing metabolite that regulates the cooperative differentiation and adaptation of fungal colonies to environmental changes [16]. Alternatively, IAA has also been proposed to be secreted by different *Saccharomyces cerevisiae* strains to inhibit growth of competing microbes and thus allow them to gain a competitive edge in specific ecological niches [17]. In sum, despite both its widespread occurrence across phylogenetically diverse fungal species, and its apparent metabolic abundance under some environmental conditions, the physiological role of IAA in fungi, whether as a secreted or an intracellular metabolite, remains largely unexplored.

The biosynthesis of IAA is best studied in plants and bacteria, which engage multiple pathways to produce IAA from tryptophan (coined Trp-dependent pathways), as well as Trp-independent pathways that remain largely elusive [2, 5]. Although phylogenetic analyses indicate that the Trp-dependent pathways of IAA biosynthesis have evolved separately in plants and bacteria [3, 18, 19], most of these pathways appear to be quite similar in these organisms. Accordingly, the major proposed Trp-dependent IAA biosynthesis pathways in plants and/or bacteria include the indole-3-pyruvic acid (IPA), the indole-3-acetamide (IAM), the indole-3-acetonitrile (IAN), the tryptamine (TAM), and the indole-3-acetaldoxime (IAOx) pathways [2, 20–22]. Notably, these major pathways are to some extent also interlinked and not all enzymatic reactions are yet fully understood. Whether fungi employ any of these known IAA biosynthesis pathways is currently not well established. The available evidence suggest, however, that fungi are able to convert Trp to IAA, likely by utilizing aromatic amino acid transferases (*e*.*g*., Tam1/2 in *Ustilago maydis* and Aro8/9 in *S*. *cerevisiae*) that metabolize Trp to IPA and cytoplasmic aldehyde dehydrogenases (*e*.*g*., Iad1/2 in *U*. *maydis* and Ald2/3 in *S*. *cerevisiae*) that reduce indole-3-acetaldehyde to IAA [11, 23–25]. Thus, the biochemical reactions leading to IAA synthesis, much like its physiological roles, remain largely mysterious in fungi.

Eukaryotic cell growth is dynamically regulated and fine-tuned by nutrient-signaling pathways among which the conserved Target Of Rapamycin Complex 1 (TORC1) pathway plays a pivotal role as it couples environmental and nutritional cues to downstream effectors that oppositely control anabolic (*e*.*g*., protein translation) and catabolic (*e*.*g*., macroautophagy) growth-related processes [26, 27]. The structure of TORC1 in its core is highly conserved among eukaryotes and consists of a dimer of a heterotrimeric complex that harbors a TOR serine/threonine protein kinase (mTOR in mammals; Tor1 or Tor2 in the budding yeast *S*. *cerevisiae*) and two regulatory proteins coined Raptor (for regulatory-associated protein of mTOR; Kog1 in yeast) and mammalian LST8 (mLST8; Lst8 in yeast) [28]. Additional seemingly non-conserved proteins such as the mammalian proline-rich Akt substrate of 40 kDa (PRAS40) and DEP domain-containing mTOR-interacting protein (DEPTOR), or budding yeast Tco89, associate with this core complex to adapt its function to species-specific requirements [29–32]. Amino acids are potent activators of TORC1 that act through the conserved Rag guanosine triphosphatases (GTPases), which form heterodimers of RagA (or RagB) and RagC (or RagD) in higher eukaryotes and Gtr1 and Gtr2 in yeast [33–35]. High levels of amino acids promote the TORC1-activating configuration of the Rag GTPases (*i*.*e*. RagA/B/Gtr1 GTP- and RagC/D/Gtr2 GDP-loaded), while amino acid starvation converts the Rag GTPases into their opposite loading state (*i*.*e*. RagA/B/Gtr1 GDP- and RagC/D/Gtr2 GTP-loaded). The Rag GTPases function within larger complexes coined Ragulator-Rag GTPase in mammals or EGO complex (EGOC) in yeast that predominately act on lysosomal/vacuolar (and yeast endosomal) surfaces where their GTP-/GDP-loading status is subjected to an elaborated network of regulatory mechanisms. These include Ragulator, which functions as a RagA/B guanine nucleotide exchange factor (GEF) [36, 37], and two highly conserved GAP complexes for RagA/B/Gtr1 and RagB/C/Gtr2 coined GATOR1/SEACIT (mammals/yeast) and FNIP-FLCN/Lst4-Lst7 (mammals/yeast), respectively [38–43]. The currently known amino-acid sensitive signaling components upstream of these Rag GTPase regulators include the vacuolar ATPase (v-ATPase) and the lysosomal amino acid transporter SLC38A9 that mediate intra-lysosomal amino acid signals to Ragulator [44–46]; the cytosolic leucine and arginine sensors Sestrin2 and CASTOR1, respectively, that impinge on GATOR1 via the conserved GATOR1-interacting GATOR2 complex (termed SEACAT in yeast) [40, 47–51]; and SAMTOR, an S-adenosylmethionine-binding protein that signals methionine abundance to GATOR1 [52]. Interestingly, all of these regulatory mechanisms implicate amino acids as positive inputs that indirectly stimulate TORC1. Whether physiologically relevant metabolites exist that inhibit TORC1 is currently not known.

Here, we confirm an earlier observation that IAA inhibits growth of *S*. *cerevisiae* when applied in the low millimolar range in the medium and show by diverse genetic means that the major growth-limiting target of IAA in yeast is TORC1. Corroborating these findings, our biochemical analyses also demonstrate that IAA inhibits TORC1 both *in vivo* and *in vitro*. Intriguingly, upon entry into stationary phase, yeast cells accumulate intracellular IAA to the extent that it is expected to contribute to metabolic downregulation of TORC1. In parallel, yeast cells also secrete IAA into the medium, but the respective levels remain by far too low to significantly impact growth as part of a quorum-sensing mechanism under the conditions studied. Thus, downregulation of TORC1, and hence proper entry of yeast cells into a quiescent state, likely relies not only on decreased amino acid-dependent stimulation by Rag-GTPases, but also on active metabolic inhibition by IAA.

## Results

### Chemical genetic profiling identifies the TORC1 pathway as potential target of IAA

**Fig 1:**
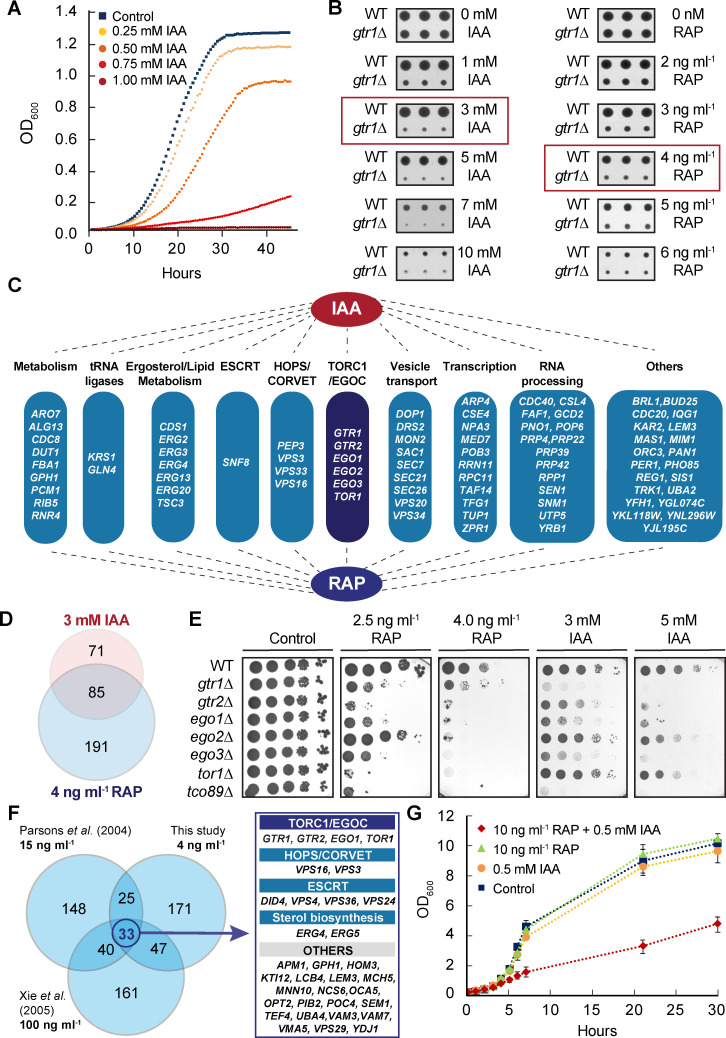
Chemical-genetic profiling identifies the TORC1 pathway as a target of IAA. (A) IAA inhibits growth of wild-type cells in liquid cultures. Growth of wild-type (BY4741) cells in the presence of the indicated concentrations of indole-3-acetic acid (IAA) was monitored using a Bioscreen C reader set at 30°C with readings (OD_600_) taken every 30 min. (B) IAA inhibits growth of wild-type cells on plates. Wild-type (BY4741) and *gtr1Δ* cells were spotted in triplicates on plates containing the indicated IAA or rapamycin (RAP) concentrations and grown for 2 days at 30°C. The red boxes indicate the sub-inhibitory drug concentrations used for the chemical-genetic profiling. (C) Chemical-genetic profiling pinpoints 85 genes, which when deleted confer both IAA- and RAP-sensitivity. The genes were clustered according to the indicated functional categories. Notably, *tco89Δ* was not recovered in this screen, likely because the knockout collection strain has a secondary suppressor mutation (K1943E) in *TOR1* [90]. ESCRT, endosomal sorting complexes required for transport; HOPS, homotypic fusion and vacuole protein sorting; CORVET, class C core vacuole/endosome tethering factor. (D) Venn diagram indicating the number of mutants from the collection of viable yeast haploid deletion mutants that are sensitive to the indicated concentrations of IAA, RAP, or IAA and RAP. (E) Loss of ECOC and TORC1 subunits causes sensitivity to both IAA and RAP. WT and indicated mutant strains (all in the BY4741 background) were grown to exponential growth phase, spotted (10-fold serial dilutions) on YPD plates containing no (control), or the indicated concentrations of rapamycin or IAA, and grown for 3 days at 30°C. (F) Venn diagram showing the overlaps between lists of mutant strains (originating from the indicated studies [54, 91]) that are sensitive to rapamycin (concentrations used are shown in bold). A total of 33 rapamycin-sensitive mutants were found in all three studies. The respective genes were functionally clustered and listed on the right. (G) IAA and rapamycin inhibit growth of wild-type cells. Growth in the presence, or not (control), of the indicated concentrations of IAA, rapamycin (RAP), or IAA and RAP combined was monitored in liquid batch cultures of wild-type (BY4741) cells (at 30°C) with OD_600_ samples taken at the indicated time points.

Although amino acids are potent activators of growth, the amino acid-derived compound IAA appears to strongly reduce growth in diverse fungi [12–14]. In line with these observations, we also found that IAA inhibited growth of our wild-type laboratory *S*. *cerevisiae* strain (BY4741) in liquid cultures in a dose-dependent manner (with a half-maximal inhibitory concentration [IC_50_] of 0.6 mM; Fig 1A), which is in a comparable range as previously reported [13, 53]. To identify the potential intracellular growth-related target(s) of IAA, we next established its chemical-genetic interaction profile by screening the collection of viable yeast haploid deletion mutants for hypersensitivity to IAA as previously described [54]. To this end, we used 3 mM IAA, which turned out to be sub-inhibitory for growth when applied to cells growing on plates (while higher IAA concentrations significantly reduced colony size on plates; Fig 1B). Intriguingly, among the 156 mutant strains that were classified as IAA-sensitive, we identified *gtr1Δ*, *gtr2Δ*, *ego1Δ/meh1Δ*, *ego2Δ*, and *ego3Δ/slm4Δ*, which are each defective for an individual subunit of the pentameric EGOC, as well as *tor1Δ* (Fig 1B and 1C). Notably, our results also confirm the earlier finding that loss of EGOC subunits rendered the respective strains hypersensitive to IAA [53]. Because these data combined indicated that IAA may potentially target the TORC1 pathway, we independently assessed the chemical genetic interaction profile of the TORC1 inhibitor rapamycin that we used at the sub-inhibitory concentration of 4 ng ml^-1^ (Fig 1B). Interestingly, of the 276 rapamycin-sensitive strains, 85 turned out to be also IAA-sensitive (Fig 1C and 1D). In other words, more than 54% of the IAA-sensitive strains were also rapamycin-sensitive and these included all the EGOC mutants and the TORC1 mutants *tor1Δ* and *tco89Δ*, as we also independently confirmed in drop spot assays (Fig 1E). As our chemical-genetic interaction profile of rapamycin overlapped even better with the one of IAA than with any of the two previously published profiles of rapamycin (Fig 1F), these data indicate that the TORC1 pathway is a major target of IAA in yeast. They do not exclude, however, that IAA may impinge on additional growth-related mechanism in parallel to the TORC1 pathway. Of note, rapamycin and IAA likely act through different mechanisms which is supported by our finding that low levels of rapamycin and IAA have little effect on growth when applied individually, but strongly reduce growth when combined (Fig 1G).

### Saturated transposon analysis in yeast corroborates that IAA targets the TORC1 pathway

**Fig 2:**
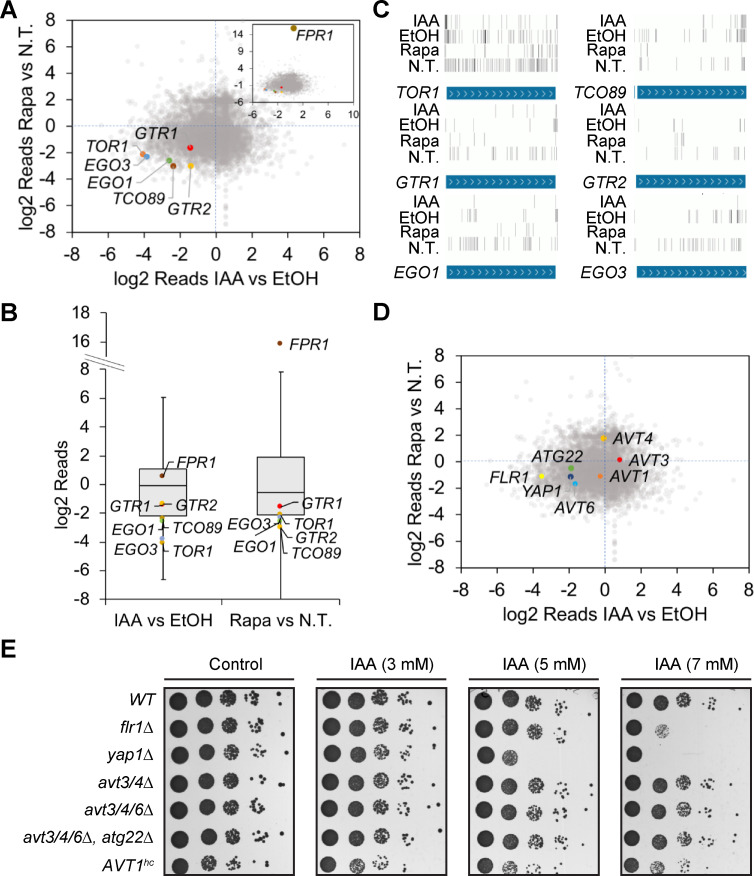
Saturated transposon analysis in yeast pinpoints the TORC1 pathway as target of rapamycin and IAA. (A) The transposition profiles of TORC1 and EGOC genes from SATAY screenings in the presence of rapamycin or IAA correlate. The dot-plot graph shows the number of transposition events (log2 transformed) in SATAY screenings (using W303-1B wild-type cells) in the presence of 1 mM IAA or 10 nM rapamycin (Rapa), normalized to the events in the presence of the respective controls. The number of transposition events in *TOR1*, *TCO89*, *GTR1*, *GTR2*, *EGO1*, and *EGO3* in the presence of IAA correlates with the one in the presence of rapamycin. *FPR1*, heavily enriched in transposons in the presence of rapamycin, is shown in a nested dot-plot for scaling reasons. N.T., non-treated control. (B) TORC1 and EGOC genes are part of the 10% that are least enriched in transposition events both in the presence of IAA and rapamycin. Box plot showing the ratio of transposition events (log2 transformed) in SATAY screenings detected in the presence of 1 mM IAA or 10 nM rapamycin versus the number of events in the presence of the respective controls. The gray boxes include all the values above the 10^th^ and below the 90^th^ percentiles and a line representing the median value. (C) Transposon insertion profiles of selected genes in the presence of 1 mM IAA, 10 nM rapamycin, and their respective controls (EtOH-treated or untreated [N.T.] WT). The position of the detected transposition events in the indicated genes are mapped along their ORFs. The intensity of the bars indicates the number of transposition events in that specific position. (D) The transposition profiles of *AVT* genes from SATAY screenings in the presence of rapamycin or IAA do not correlate. Dot-plot graph showing the number of transposition events (log2 transformed) in SATAY screenings in the presence of 1 mM IAA or 10 nM rapamycin, normalized to the events in the presence of the respective controls. The number of transposition events in *AVT1*, *AVT3*, *AVT4*, *AVT6*, or *ATG22* in the presence of IAA does not correlate with the one in the presence of rapamycin. *YAP1* and *FLR1* are protected from transposition events in the presence of IAA, but not rapamycin. (E) Drop spots of selected mutants on rapamycin- and IAA-containing plates. Drop spots (10-fold dilutions of 1 OD_600_/ml cell suspensions) of the indicated prototrophic (BY4741 background) strains were performed on SD medium (devoid of leucine, histidine, uracil, tryptophan, and adenine) with increasing concentrations of IAA. *yap1Δ* and *flr1Δ* cells show IAA sensitivity, while *avt3/4Δ*, *avt3/4/6Δ*, and *avt3/4/6Δ atg22Δ* cells, and cells overexpressing *AVT1* from a high-copy plasmid (*AVT1*^*hc*^), do not show altered growth on IAA.

To corroborate our chemical-genetic profile data, we decided to use SAturated Transposon Analysis in Yeast (SATAY), which is particularly suited to discover loci that are important for growth under defined conditions and can hence be used to identify drug targets [55]. We grew a 57 million-clones transposon library for ~7 generations in the absence or presence of 1 mM IAA, and compared the ratio of reads obtained for each coding sequence in the presence of IAA or in the presence of rapamycin versus untreated libraries [55, 56]. Strikingly, the components of the EGOC and TORC1 complexes were significantly underrepresented in both cases (Fig 2A, 2B and 2C), indicating that cells incapable of expressing these genes were hypersensitive to both IAA and rapamycin. In particular, genes of the EGOC (*EGO1* and *EGO3*) or TORC1 (*TOR1* and *TCO89*) were part of the most underrepresented 10% in both treatments (Fig 2B).

Many genes in the two datasets were, however, not evidently correlated, which highlights the specificities of the different treatments. This can be explained by the mechanisms by which rapamycin and IAA impact on cell growth. For instance, *FPR1*, the product of which specifically binds rapamycin to inhibit TORC1 [57], is highly represented in the rapamycin-treated, but not in the IAA-treated library, which is consistent with rapamycin resistance being mediated through *fpr1* deficiency [58] (Fig 2A and 2B). In contrast, transpositions within two genes, *FLR1* and *YAP1*, specifically caused IAA sensitivity (Fig 2D and 2E). Flr1 is a drug transporter at the plasma membrane and Yap1 an oxidation-sensitive transcription factor responsible for stress-induced *FLR1* expression [56]. In the presence of high amounts of extracellular IAA, Yap1-deficient cells therefore likely accumulate more IAA intracellularly because they fail to activate *FLR1* transcription. Consistent with this assumption, both *yap1Δ* and *flr1Δ* strains not only exhibited IAA-sensitive growth (Fig 2E), but also accumulated significantly higher levels of IAA than wild-type cells when challenged for 24 h with 2 mM extracellular IAA (*i*.*e*. 4.9 ± 2.1 mM for wild-type, 7.1 ± 1.5 mM for *yap1Δ*, and 16.9 ± 3.6 mM for *flr1Δ* cells). Interestingly, a previous SATAY screening using an auxin-inducible degron also found that transposons within *FLR1* and *YAP1* caused cell death in IAA-treated conditions. In this case, IAA caused toxic degradation of the DNA repair factor Tdp1 [59]. Together with our current data, these findings suggest that Flr1 is necessary for efficient IAA efflux out of the cells, explaining why transposons in this gene and its transcriptional regulator are specifically detrimental for fitness when cells are grown in the presence of IAA, but not when they are grown in the presence of low rapamycin concentrations.

Of note, the IAA sensitive growth of *yap1-1* mutants has previously been reported to result from the upregulation of the amino acid permeases Avt3 and Avt4 that were proposed to mediate IAA transport across the plasma membrane [13]. However, at variance with this report, several studies have meanwhile shown that Avt3 and Avt4 are primarily expressed at the vacuolar membrane where they transport glutamine, leucine, isoleucine, asparagine, tyrosine, and proline from the vacuoles into the cytoplasm [60–63]. In addition, both our SATAY analyses and our drop spot analyses were congruent in that they did not reveal any changes in IAA-sensitivity for the single *avt3Δ* or *avt4Δ* strains (Fig 2D and 2E). Similarly, simultaneous loss of both Avt3 and Avt4, even when combined with loss of the partially redundant vacuolar amino acid effluxer Atg22 or the closely related vacuolar amino acid permease Avt6 [64, 65], did not alter IAA-sensitive growth (Fig 2E). Finally, overexpression of Avt1, an amino permease that mediates vacuolar uptake of several amino acids [61, 66], caused a slight slow-growth phenotype, but had also no significant effect on IAA-sensitivity (Fig 2E). All of these data combined therefore suggest that the IAA-sensitivity of *yap1* mutants results primarily from a failure to stimulate IAA efflux through Flr1, rather than from an enhanced uptake of IAA through Avt3 and/or Avt4.

### IAA inhibits TORC1 *in vivo* and *in vitro*

**Fig 3:**
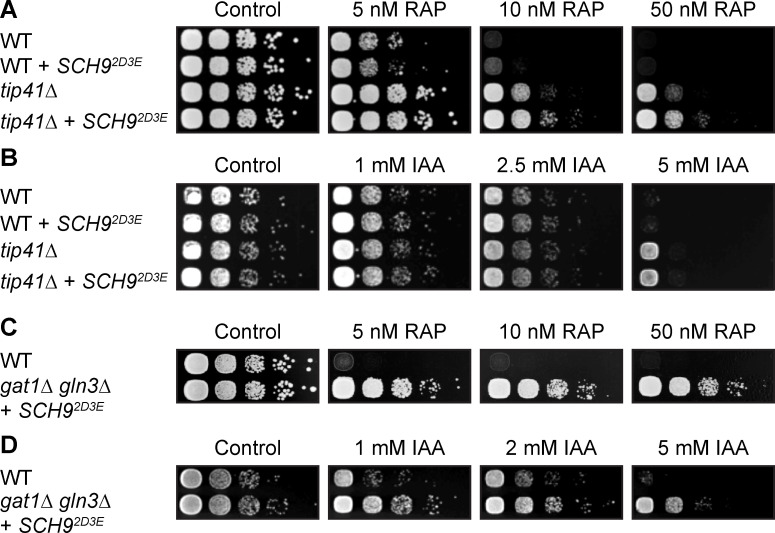
TORC1 bypass mutations confer IAA resistance. (A-D) Yeast cells can be genetically engineered to bypass the essential functions of TORC1. Spotting 10-fold serial dilutions of these cells onto YPD plates containing drug vehicle or the indicated concentration of rapamycin (RAP; [A] and [C]) or IAA ([B] and [D]) shows that TORC1 bypass (*tip41Δ SCH9*^*2D3E*^ [A] and [B]; strains in BY4741 background), or *gat1Δ gln3Δ SCH9*^*2D3E*^ ([C] and [D]; strains in TB50a background) confers resistance to both of these compounds.

To study whether TORC1 is the primary growth-limiting target of IAA in yeast, we used yeast strains that are able to bypass the essential function of TORC1. Such strains can be engineered by combining the constitutively active TORC1 effector Sch9^2D2E^ with loss of either Tip41 [67], or loss Gat1 and Gln3 combined [68–70]. Both TORC1 bypass strains (*i*.*e*. *tip41Δ SCH9*^*2D3E*^ and *gat1Δ gln3Δ SCH9*^*2D3E*^), but not wild-type cells, were able to grow on plates containing high doses of rapamycin or IAA (Fig 3A, 3B, 3C and 3D). These data also fit well with our results from a non-saturated screen for mutations that enable better growth of wild-type cells in the presence of 5 mM IAA. Accordingly, whole-genome sequencing (performed as previously described in [71]) allowed us to identify 2 mutations that fulfilled the criteria of our screening procedure, namely a stop codon right after Tyr^244^ in Tip41 and a frame-shift mutation after Ser^193^ in Gat1. In sum, we infer form our genetic and biochemical analyses that TORC1 is the major growth-limiting target of IAA in yeast.

**Fig 4:**
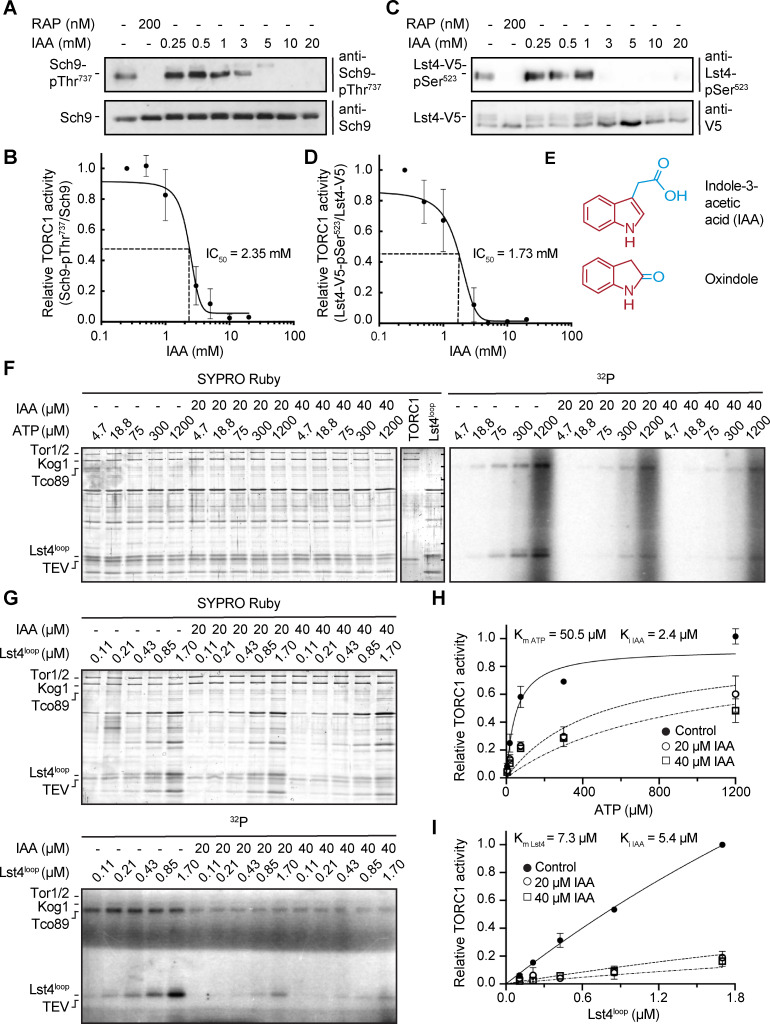
IAA inhibits TORC1 *in vivo* and *in vitro*. (A-D) IAA inhibits TORC1 *in vivo*. TORC1 activity was assessed *in vivo* by measuring the phosphorylation level of the TORC1 target sites Thr^737^ in Sch9 (A) and Ser^523^ in Lst4 (C) using phospho-specific anti-Sch9-pThr^737^ and anti-Lst4-pSer^523^ antibodies, respectively, in cells (BY4741 background) treated for 10 min with the indicated concentrations of IAA or rapamycin (RAP). The relative TORC1 activities in (A) and (C) were quantified and plotted in (B) and (D), respectively, to assess the respective IC_50_ values. All assay points were done in triplicates and expressed as means ± SD. (E) Chemical structure of indole-3-acetic acid (IAA) and oxindole. The indole core is marked in red. (F, G) IAA inhibits TORC1 *in vitro*. TORC1 was purified from yeast and its kinase activity was measured as a function of increasing ATP concentrations (in the presence of 400 ng of the purified, recombinant His_6_-Lst4^loop^ substrate that encompasses the intra DENN loop (*i*.*e*. amino acids 200–400 of) of Lst4 (Péli-Gulli et al., 2017) (F), or increasing amounts of His_6_-Lst4^loop^ (in the presence of 300 μM ATP) (G), and varying concentrations of IAA (*i*.*e*. 0, 20, or 40 μM). Representative SYPRO Ruby staining and autoradiography blots (^32^P; with phosphorylated Lst4 and Tco89, a TORC1 subunit that is targeted by TORC1 itself and also serves as a proxy for TORC1 activity [92]) are shown in (F) and (G). In the central panel in (F), purified TORC1 and His_6_-Lst4^loop^ preparations were loaded separately as controls, with horizontal lines indicating the molecular weight markers that correspond (from top to bottom) to 250-, 130-, 100-, 70-, 55-, 35-, and 25 kDa. TEV denotes the Tobacco Etch Virus protease that was used to release TORC1 from beads. (H, I) The relative TORC1 activities in (F; towards Tco89) and (G; towards Lst4) were quantified and plotted in (H) and (I), respectively. Curve fittings and calculations of K_m_/K_i_ values were carried out with the GraphPad PRISM software. All assay points were done in triplicates and expressed as means ± SD.

To extend our genetic observations, we next asked whether IAA may inhibit TORC1 *in vivo*. When applied to cells, IAA, like rapamycin [68], caused a dose-dependent dephosphorylation of two independent *bona fide* TORC1 target residues, namely Thr^737^ in Sch9 and Ser^523^ in Lst4 [69, 72] (Fig 4A, 4B, 4C and 4D). These findings are also in line with the observation that IAA (4 mM), like rapamycin, strongly decreased and increased the phosphorylation of the distal TORC1 targets Rps6 and Par32, respectively [53, 73, 74]. The half maximal inhibitory concentrations (IC_50_) for IAA-mediated TORC1 inhibition were 2.35 mM and 1.73 mM when assayed using pThr^737^ and pSer^523^ in Sch9 and Lst4, respectively. These values are slightly higher but match reasonably well with the IC_50_ for IAA-mediated growth inhibition.

Oxindole, which is structurally very similar to IAA (Fig 4E), and oxindole-based derivatives have been broadly used as protein kinase inhibitors in cancer therapies ([75]. Both crystallography and molecular modeling studies have shown that the oxindole core forms hydrogen bonds with the backbone of the hinge region within protein kinases that normally accommodates the adenine ring of ATP, thereby acting as a type I ATP-competitive protein kinase inhibitor [76, 77]. The structural resemblance of oxindole and IAA therefore raised the possibility that IAA may inhibit TORC1 directly through competition for ATP. Classical assays of enzyme kinetics using TORC1 purified from yeast demonstrated that this was indeed the case (Fig 4F and 4G). Accordingly, based on regression analyses carried out with the GraphPad Prism curve fitting program, the behavior of IAA matched best with that of a competitive TORC1 inhibitor with respect to ATP and a non-competitive inhibitor with respect to its physiological substrate Lst4 (Fig 4F, 4G, 4H and 4I and S1 Data).

### Stationary phase entry triggers intracellular accumulation IAA

**Fig 5:**
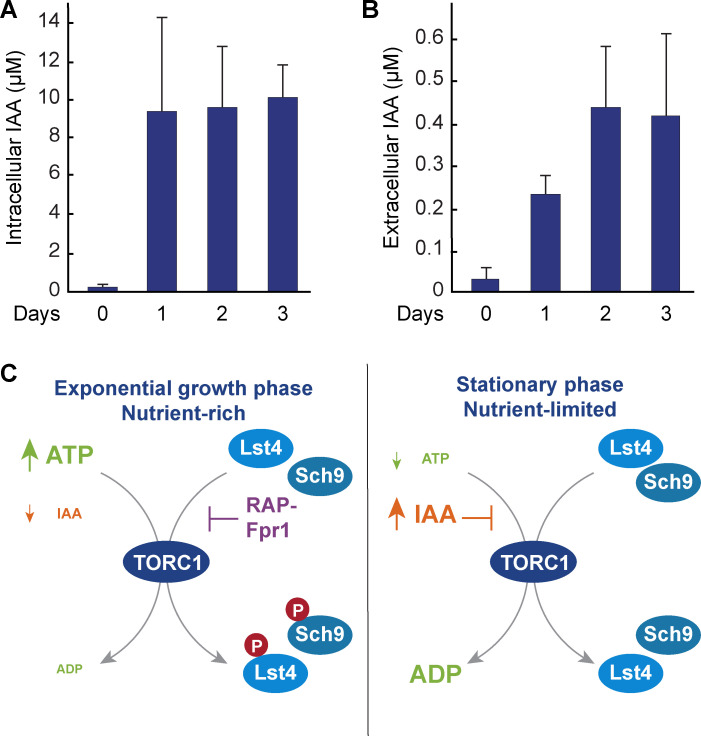
IAA accumulates in stationary phase cells. (A, B) The intracellular (A) and extracellular (B) concentrations of IAA (n = 3; +SD) were determined in exponentially growing (BY4741) wild-type cells (0) and in cells grown to stationary phase for the indicated number of days. (C) Indole-3-acetic acid (IAA) acts as an ATP-competitive inhibitor of TORC1 in nutrient-starved stationary phase cells. In cells growing exponentially on nutrient-rich media, ATP levels are high (about 3mM; [82]) and IAA level are low (< 0.05 μM; [83]). Under these conditions, TORC1 is able to phosphorylate substrates such as Lst4 and Sch9 [69, 72]. In complex with Fpr1, rapamycin binds to the FRB domain near the entrance of the catalytic cleft of TORC1, thereby interfering with the recruitment of substrates to the active site [93]. In stationary phase cells, intracellular ATP levels drop below 56 μM [82, 83], while the ones of IAA increase up to 10 μM (Fig 5A), which, based on the kinetic parameters elaborated here (see text for details), would enable IAA to efficiently compete for ATP and thereby contribute to TORC1 inhibition.

While our studies so far relied on the administration of IAA to the extracellular medium, we wondered whether the biosynthesis of IAA may have any physiological bearing in yeast. To this end, we determined both intra- and extracellular IAA levels in exponentially growing and nutrient-limited yeast cells entering stationary phase. In exponentially growing cultures, cells contained very low levels of intracellular IAA (< 0.2 μM), which was matched with also very low levels of IAA in the extracellular space (< 0.04 μM; Fig 5A and 5B). Intriguingly, however, when cells entered stationary phase, they accumulated considerable amounts of intracellular IAA (up to 10 μM; Fig 5A) and appeared to also secrete substantial amounts of IAA (up to 0.4 μM) into the medium (Fig 5B). In parallel to the inhibition of TORC1 by oligomerization into structures coined TOROIDS [78], IAA may therefore contribute to the inactivation of TORC1 as a metabolic cue when cells enter stationary phase.

## Discussion

The role of IAA as an intracellular and/or secreted extracellular fungal metabolite has hitherto largely remained enigmatic. All of our genetic, biochemical and physiological data combined indicate that IAA accumulates intracellularly when cells approach stationary phase and may then serve to inhibit growth by acting as an ATP-competitive inhibitor of TORC1. One caveat of this model, however, is that type I inhibitors like IAA are expected to invariably occupy the adenine-binding region in most protein kinases [77], which raises the question of whether TORC1 exhibits characteristic qualities that make it more sensitive to IAA than most other protein kinases. One possibility, in this context, is that the acetic acid side chain of the indole core may provide functionality towards additional regions in the kinase domain of TORC1 and thereby form the basis for inhibitor selectivity [76]. Perhaps more conspicuously, however, TORC1 displays a rather high K_m_ for ATP (*i*.*e*. > 1 mM for mammalian TORC1 [79], or 50.5 μM for yeast TORC1; Fig 4H) when compared to the ones of most other protein kinases that lie in the low micromolar range [80]. This is relevant because the potency (IC_50_) of a given reversible ATP competitor critically depends on its affinity (K_i_), the ATP concentration ([ATP]), and the K_m_ for ATP (K_m ATP_) as defined by the Cheng-Prusoff equation (IC_50_ = K_i_*(1 + [ATP]/K_m ATP_); [81]). In other words, among a set of kinases for which it exhibits a similar K_i_, a competitive inhibitor most stringently inhibits those with the highest K_m ATP._ Another important corollary of this equation is that the IC_50_ for IAA-mediated TORC1 inhibition relies on the intracellular [ATP]. In exponentially growing yeast cells, the [ATP] is approximately 3 mM [82], which, given a K_i IAA_ of 2.4 μM and a K_m ATP_ of 50.5 μM for TORC1 (Fig 4H), results in an IC_50 IAA_ value of 145 μM. In such cells, the minute intracellular IAA levels (< 0.2 μM) would obviously not be able to measurably restrain TORC1 activity. Intriguingly, however, when cells reach the 3^rd^ day in stationary phase, the [ATP] drops more than 50-fold (to about 56 μM) [83], which would reduce the IC_50 IAA_ to 5.1 μM and enable the intracellular IAA (about 10 μM: Fig 5A) to inhibit TORC1 by 66.2% (according to the equation: % inhibition = [IAA]/([IAA] + IC_50 IAA_)). Thus, the specific characteristics of TORC1, coupled with a dramatic drop in ATP levels, are likely to assign the rising IAA levels a significant role in TORC1 inhibition when cells enter stationary phase (Fig 5C). We deem it therefore likely that IAA is a physiologically relevant compound that serves to contribute to metabolically locking TORC1 in an inactive state to ensure proper establishment of the quiescence program. Building on these findings, it will be important to identify the biochemical reactions and the respective genes that lead to IAA biosynthesis in order to be able to genetically corroborate our model. Equally important, pinpointing permeases that may shuffle IAA between the cytoplasm and the vacuole or the extracellular space should provide means to characterize and modulate the intracellular distribution of IAA and thus help delineate the relative importance of IAA in TORC1 inhibition in the future.

With respect to the role of IAA as an extracellular metabolite, circumstantial evidence suggested that IAA secretion may either serve to cooperatively control physiological responses to changing environmental conditions within yeast cultures [16], or to inhibit growth of competing fungi in specific ecological niches [17]. Our study indicates that *S*. *cerevisiae* does indeed secrete IAA into the extracellular space and that the respective concentrations can reach up to 0.5 μM in stationary phase batch cultures. These levels are, however, roughly 100-fold lower than the ones needed to induce filamentous growth [13], and more than 1000-fold lower than the ones needed to attain 50% growth reduction in exponentially growing wild-type cells (IC_50_ of 0.6 mM; Fig 1A). In our batch cultures, IAA secretion is therefore unlikely to coordinate aspects of growth as part of a presumed quorum-sensing mechanism. Nevertheless, to what extent extracellular IAA may impact on the physiology of individual yeast cells within a culture conceivably also depends on the relative expression of permeases that import or export IAA across the plasma membrane, a parameter that may also strongly vary between exponentially growing and stationary phase cells. In addition, our studies do currently also not exclude the possibility that the levels of IAA secreted by yeast cells growing in micro-niches in the wild may attain higher concentrations that may be relevant for quorum-sensing mechanisms and/or inhibition of other microbial species (to provide budding yeast a competitive edge under specific conditions). Concluding assessment of these issues will therefore not only require further studies that are aimed at the identification and characterization of the entire compendium of IAA permeases in yeast, but also the elaboration of more sophisticated experimental approaches that mimic a competitive setting of microbial interactors in the wild. In this context, we would also like to point out that the extracellular IAA levels that we determined in stationary phase batch cultures are also about 1000-fold lower than those reported previously for various *S*. *cerevisiae* strains including S288C [17], which is closely related to the BY4147 strain used here [84]. Two factors may contribute to the observed discrepancy between our study and the one from Liu and co-workers. Firstly, Liu et al. used the Salkowski colorimetric assay, which is less specific than the liquid chromatography-mass spectrometry (LC-MS) protocol we employed here, because it is unable to discriminate between various indolic compounds (*e*.*g*., IAA, IPA, and IAM [85]) and may hence tend to overestimate IAA levels. Secondly, in the latter study, the authors supplemented the growth medium with high amounts of the IAA precursor L-tryptophan (*i*.*e*. 0.1% or 4.8 mM), which boosts the biosynthesis and possibly secretion of IAA. A role, if any, of secreted IAA in defining the competition among different yeast species that occupy the same niche may therefore specifically depend on high extracellular L-tryptophan levels.

A final point that we would like to highlight is that our findings have also important repercussions for experimental approaches that use auxin-induced degrons (AID) to study the function of individual proteins. Accordingly, such studies are often carried out with IAA levels in the range that we found here to inhibit TORC1. This caveat can be addressed by the inclusion of proper controls (*e*.*g*., IAA-treated cells devoid of the AID degron). In those cases where the AID degron is used in combination with a SATAY screening, our current data may serve as a baseline to mitigate this problem.

## Materials and methods

### Yeast strains, plasmids, and growth conditions

*Saccharomyces cerevisiae* strains and plasmids are listed in S1 and S2 Tables, respectively. Unless stated otherwise, the strains were made prototrophic by transformation with the required empty centromeric plasmids listed in S2 Table. Yeast cells were pregrown in synthetic dropout (SD; 0.17% yeast nitrogen base, 0.5% ammonium sulfate [AS], 0.2% dropout mix [USBiological], and 2% glucose) medium to maintain plasmids. The following day, the cultures were diluted in synthetic complete medium (SC; SD with all amino acids).

### Saturated transposon assay in yeast (SATAY)

The 57 million independent transposed clones library generated in the W303 background described in [86] was frozen for further usage following induction of the transposition and prior to regrowth in selective medium. The library (6 ml) was thawed to inoculate a 2 x 2L SD-ADE 2% glucose culture at OD_600_ of 0.176. Plating of the inoculum on non-selective (SD) and selective (SD-ADE) media allowed us to estimate that 3E+07 independent transposed clones were present that represented 12.6% of the total number of cells. The culture was incubated at 30°C and shaken at 160 rpm for 46 hours in a Multitron Standard Infors incubator. The same instrument was used for all further incubations. Plating of the saturated culture on selective and non-selective media indicated that the transposed population represented over 82% of the cells. Plating also allowed to estimate that each transposed cell initially inoculated, had undergone 12.7 cell cycles and was represented by 7E+03 copies. The saturated culture (65.3 ml) was then further inoculated in 3.2 L of prewarmed SD-ADE 2% glucose and incubated for 5 hours at 30°C, shaking at 160 rpm. Plating of the culture indicated that the transposed cells had undergone 2.44 generations, therefore were in exponential phase and ready to be treated with IAA. The culture was split in 3 x 500 ml samples, each containing 3.48+09 cells, of which 3.03E+09 were transposed. A freshly prepared IAA solution (778 nM in 100% EtOH) was added to a final concentration of 1 mM or 1.5 mM IAA. 100% EtOH (1.285 ml) was added to form a NO IAA control. The volume of 100% EtOH was adjusted to each IAA containing flask to ensure it was kept constant across samples. The cultures were incubated at 30°C, shaking at 160 rpm. After 18 hours of incubation, which corresponded to 2 generations of the transposed cells grown in presence of 1.5 mM IAA and 2.5 generations of the transposed cells grown in presence of 1 mM IAA or in the absence of IAA, as assessed by plating of the cultures on selective and non-selective media, the cultures were further diluted in 500 ml prewarmed SD-ADE 2% glucose and re-exposed to the same concentrations of IAA. Dilutions (38- to 54-fold) of the cultures treated first were used to ensure that the same amount of transposed cells received the second IAA treatment. Plating of each individual culture at T0 of the second IAA treatment confirmed that each sample contained 3.43E+08 ADE+ cells (No IAA), 4.48E+08 ADE+ cells (1 mM IAA), and 3.56E+08 ADE+ cells (1.5 mM IAA). The cultures were incubated for at 30°C, shaking at 160 rpm. Aliquots of the cultures were simultaneously grown in a Labtech International Ltd Bioscreen C instrument and the doubling times were calculated to be 2.05h (NO IAA), 2.28h (1 mM IAA), and 2.62h (1.5 mM IAA). The NO IAA- and 1 mM IAA-treated cultures were harvested after growing for 18 hours and 45 min, which corresponded to an additional 5.46 and 4.17 generations, respectively.

Genomic DNA was prepared and treated as previously described [55], with the exception of the PCR, which was performed using NEB *Taq* DNA Polymerase with ThermoPol Buffer (175 units per sub-library– 1 min 95°C, [30 sec 95°C, 30 sec 55°C, 3 min 68°C] x 35, 10 min 68°C) and indexed primers (S3 Table). After PCR cleanup, libraries were quantified using a nanodrop, pooled to equal concentrations and sequenced at the Oxford Zoology Sequencing Facility on an Illumina NextSeq550 instrument, using a NextSeq 500/550 High Output Kit v2.5 (75 Cycles). The sequencing reads were aligned to the yeast genome. A total of 559039, 357008, and 616112 independent transposons were mapped in the NO IAA, 1 mM IAA, and 1.5 mM IAA libraries, respectively. The libraries characteristics are indicated in S4 Table. Data are accessible at http://genome-euro.ucsc.edu/s/AgnesHM/Nicastro_et%20al_2020_Transposons, where IAA indicates the library treated with 1mM IAA, EtOH, the same library but mock-treated, rapamycin, a previously described rapamycin-treated library, and N.T., its non-treated control (Michel et. al 2017). Six other previously described libraries (Michel et. al 2017) were added for the purpose of comparison.

### Chemical-genetic screening

The screening was carried out using a robot apparatus (Singer Instruments) and the knock out yeast collection (384 wells, Euroscarf) spotted in quadruplicate on synthetic medium plates depleted of amino acids, in presence of the vehicle (90% ethanol/10% Tween) or the drug (3 mM IAA or 4 nM rapamycin). The plates were analyzed using the Rothstein lab platform (http://www.rothsteinlab.com). The mutants with a z-score ≥ 1.88 were considered sensitive to the treatment (S5 Table and S1 Data).

### *In Vivo* TORC1 kinase assays

*In vivo* TORC1 activity was assayed as previously described [43, 72], using phosphospecific anti-Sch9-pThr^737^ and anti-Lst4-pSer^523^ antibodies, and anti-Sch9 and anti-V5 antibodies (GenScript) to probe endogenous Sch9 and plasmid-encoded Lst4-V5, respectively.

### TORC1 purification

TORC1 was purified as described in [72], with minor modifications. A Tco89-TEV-TAP-expressing yeast strain was grown to late exponential growth phase in YPD, treated with extra YPD powder (50 g L^-1^) for 1 hour and afterwards for 10 min with cycloheximide (12.5 μg ml^-1^ final). The cells were collected by filtration and rapidly frozen in liquid nitrogen and subjected to cryogenic disruption with an MM 400 Mixer Mill (Retsch). The obtained frozen yeast powder was resuspended in extraction buffer (50 mM HEPES/NaOH [pH 7.5], 5 mM CHAPS, 400 mM NaCl, 1 mM EDTA, 0.5 mM DTT, 400 mM Pefabloc, and Roche complete protease inhibitor EDTA-free). The cleared lysate was incubated with IgG-coupled Dynabeads (Dynabeads M-270 Epoxy; Invitrogen) for 2 hours at 4°C. After 5 washes with wash buffer (50 mM HEPES/NaOH [pH 7.5], 5 mM CHAPS, 400 mM NaCl, and 0.5 mM DTT) the TORC1 complex was eluted using TEV protease (2% final) and stored at -80°C after addition of 10% glycerol.

### *In vitro* TORC1 kinase assays

*In vitro* kinase reactions were performed as in [72], with minor modifications. The reactions were carried out in kinase buffer (50 mM HEPES/NaOH [pH 7.5], and 150 mM NaCl), with the indicated amount of purified His_6_-Lst4^loop^ and 60 ng TORC1 (quantified with respect to the Tor1 subunit) in 30 μl total volume and started by adding the ATP Mix (4.2 mM MgCl_2_, [γ-^32^P]-ATP [Hartmann Analytic, SRP-501], and the indicated amount of ATP) and stopped by adding SDS-PAGE sample buffer. Proteins were separated by SDS-PAGE, stained with SYPRO Ruby (Sigma) to assess loading, and analyzed using a phosphoimager (Typhoon FLA 9500; GE Healthcare).

### Detection of IAA

For auxin extraction, yeast cells were incubated in methanol and shaken at 37°C for 45 min in the presence of 10 pmol d5-IAA as internal standard. After centrifugation, the pellet was re-extracted with methanol. Both supernatants were combined and dried completely. Culture media (5 ml) were acidified with formic acid (final concentration 0.5%) and extracted twice with the same volume of ethyl acetate in the presence of 50 pmol d5-IAA as internal standard. Both ethyl acetate phases were combined and dried completely. The residuals were solubilized in 0.1% acetic acid and loaded on a self-packed C18-micro-SPE column (2.5 mg, 3 μm, Dr. Maisch). Columns were washed with 0.1% acetic acid and IAA was eluted twice with 50 μl 0.5% acetic acid in acetonitrile. Eluents were dried completely and resuspended in HPLC mobile phase A (0.1% formic acid in water). LC-MS measurements were performed on a QExactive Plus mass spectrometer (Thermo Fisher) coupled to an EasyLC 1000 nanoflow-HPLC. HPLC-column tips (fused silica) with 75 μm inner diameter were self-packed with Reprosil-Pur 120 C18-AQ material (1.9 μm, Dr. Maisch) to a length of 20 cm. Analytes were separated with a gradient of A and B (0.1% formic acid in 80% acetonitrile in water) with increasing organic proportion (loading of sample with 0% B, separation ramps with a flow rate of 250 nl min^-1^: from 5–12% B within 1 min, from 12–50% B within 9 min, from 50–100% B within 1 min, hold 100% B for 18 min). The mass spectrometer was operated in positive ion mode (ESI) with an electron spray voltage of 2.3 kV at 250°C of the heated capillary temperature. Precursor-to-product ion transitions of m/z 176.07 → 130.07 for IAA, m/z 181.1→ 135.1 for d5-IAA were used as quantifier for parallel reaction monitoring (PRM) with a normalized collision energy of 30%, resolution of 35’000, AGC target value of 200’000, isolation window of 1.6 m/z and a maximum injection time of 50 ms. Absolute quantifications were done using Skyline Software [87] and a 6-point calibration curve done in parallel with sample extraction. To calculate the intracellular concentration of IAA, we assumed that the average exponentially growing yeast cell has an approximate volume of 4 fL [88], while G_1_-arrested cells (days 1–3 in Fig 5) were assumed to exhibit a 50% reduced cell volume [89].

## Supporting information

S1 Table Strains used in this study. (DOCX)

S2 Table Plasmids used in this study. (DOCX)

S3 Table Primers used in the SATAY screening. (DOCX)

S4 Table Libraries characteristics. (DOCX)

S5 Table Chemical genetic screening results. (XLSX)

S1 Data Original data sheets. (XLSX)
