# Supplementary material for: Indole-3-acetic acid is a physiological inhibitor of TORC1 in yeast
Source: PLoS Genet. 2021 Mar 9;17(3):e1009414. doi: 10.1371/journal.pgen.1009414 (PMC7978357; doi:10.1371/journal.pgen.1009414)
Supplement: S1 Table — (DOCX) [file pgen.1009414.s001.docx]

**S1 Table. Strains used in this study.**

| **Strain** | **Genotype** | **Source** | **Figure** |
| --- | --- | --- | --- |
| BY4741 | *MAT***a***;* *his3∆1, leu2∆0, met15∆0, ura3∆0* | Euroscarf | 1A, B; 3A, B; 5A, B |
| *gtr1∆* | [BY4741] *MAT***a***; gtr1∆::kanMX* | Euroscarf | 1B |
| *flr1∆* | [BY4741] *MAT***a***; flr1∆::kanMX* | Euroscarf | 2E |
| *yap1∆* | [BY4741] *MAT***a***; yap1∆::kanMX* | Euroscarf | 2E |
| *tip41∆* | [BY4741] *MAT***a***; tip41∆::kanMX* | Euroscarf | 3A, 3B |
| YL515 | [BY4741] *MATα; his3∆1, leu2∆0, ura3∆0* | [[1](#_ENREF_1)] |  |
| YL516 | [BY4741] *MAT***a***; his3∆1, leu2∆0, ura3∆0* | [[1](#_ENREF_1)] | 1E; 1G; 2E; 4A |
| MB32 | [YL516] *MAT***a***; gtr1∆::kanMX* | [[1](#_ENREF_1)] | 1E |
| NIC090 | [YL516] *MAT***a***; gtr2∆::kanMX* | This study | 1E |
| MB25 | [YL515] *MATα; ego1∆::HIS3* | [[1](#_ENREF_1)] | 1E |
| NP52-2A | [YL515] *MATα; ego2∆::HIS3* | [[2](#_ENREF_2)] | 1E |
| MB26 | [YL515] *MATα; ego3∆::HIS3* | [[1](#_ENREF_1)] | 1E |
| MP10-6C | [YL515] *MATα; tor1∆::kanMX* | This study | 1E |
| RKH311 | [YL516] *MAT***a***; tco89∆::kanMX* | [[3](#_ENREF_3)] | 1E |
| CDV312-3D | [YL516] *MAT***a***; avt3∆::kanMX avt4∆::kanMX* | This study | 2E |
| CDV313-12C | [YL515] *MAT*α*; avt3∆::kanMX avt4∆::kanMX avt6∆::kanMX* | This study | 2E |
| GB1648-4D | [YL515] *MAT*α*; avt3∆::kanMX avt4∆::kanMX avt6∆::kanMX atg22∆::kanMX* | This study | 2E |
| MP347-1B | [YL516] *MAT***a***; lst4∆::kanMX* | This study | 4C |
| TB50a | *MAT***a***; trp1, his3, ura3, leu2, rme1* | [[4](#_ENREF_4)] | 3C, D |
| TB105-3B | [TB50a] *MAT***a***; gln3∆::kanMX gat1∆::HIS3* | [[5](#_ENREF_5)] | 3C, D |
| RL170-2c | [TB50a] *MAT***a***; TCO89-TAP::TRP1* | [[6](#_ENREF_6)] | 4F-I |
| ByK911 | [W303-1B] *MAT***a***; ura3-1 leu2-3,112 his3-11,15 trp1-1 ade2-1* | [[7](#_ENREF_7)] | 2A-D |

**References**

**1**. Binda M, Péli-Gulli MP, Bonfils G, Panchaud N, Urban J, Sturgill TW, et al. The Vam6 GEF controls TORC1 by activating the EGO complex. Mol Cell. 2009; 35: 563-573. <https://doi.org/10.1016/j.molcel.2009.06.033> PMID: 19748353

**2**. Powis K, Zhang T, Panchaud N, Wang R, De Virgilio C, Ding J. Crystal structure of the Ego1-Ego2-Ego3 complex and its role in promoting Rag GTPase-dependent TORC1 signaling. Cell Res. 2015; 25: 1043-1059. <https://doi.org/10.1038/cr.2015.86> PMID: 26206314

**3**. Hatakeyama R, Péli-Gulli MP, Hu Z, Jaquenoud M, Garcia Osuna GM, Sardu A, et al. Spatially distinct pools of TORC1 balance protein homeostasis. Mol Cell. 2019; 73: 325-338 e328. <https://doi.org/10.1016/j.molcel.2018.10.040> PMID: 30527664

**4**. Beck T, Hall MN. The TOR signalling pathway controls nuclear localization of nutrient-regulated transcription factors. Nature. 1999; 402: 689-692. <https://doi.org/10.1038/45287> PMID: 10604478

**5**. Wanke V, Cameroni E, Uotila A, Piccolis M, Urban J, Loewith R, et al. Caffeine extends yeast lifespan by targeting TORC1. Mol Microbiol. 2008; 69: 277-285. <https://doi.org/10.1111/j.1365-2958.2008.06292.x> PMID: 18513215

**6**. Shimada K, Filipuzzi I, Stahl M, Helliwell SB, Studer C, Hoepfner D, et al. TORC2 signaling pathway guarantees genome stability in the face of DNA strand breaks. Mol Cell. 2013; 51: 829-839. <https://doi.org/10.1016/j.molcel.2013.08.019> PMID: 24035500

**7**. Michel AH, van Schie S, Mosbach A, Scalliet G, Kornmann B. Exploiting homologous recombination increases SATAY efficiency for loss- and gain-of-function screening. 2020. <https://doi.org/10.1101/866483>
