# Supplementary material for: Indole-3-acetic acid is a physiological inhibitor of TORC1 in yeast
Source: PLoS Genet. 2021 Mar 9;17(3):e1009414. doi: 10.1371/journal.pgen.1009414 (PMC7978357; doi:10.1371/journal.pgen.1009414)
Supplement: S2 Table — (DOCX) [file pgen.1009414.s002.docx]

**S2 Table. Plasmids used in this study.**

| **Plasmid** | **Genotype** | **Source** | **Figure** |
| --- | --- | --- | --- |
| pRS413 | *CEN, ARS, HIS3* | [[1](#_ENREF_1)] | 1E; 1G; 2E; 4A; 3C, D |
| pRS414 | *CEN, ARS, TRP1* | [[1](#_ENREF_1)] | 3C, D |
| pRS415 | *CEN, ARS, LEU2* | [[1](#_ENREF_1)] | 1E; 1G; 2E; 4A; 3A-D |
| pRS416 | *CEN, ARS, URA3* | [[1](#_ENREF_1)] | 1E; 1G; 2E; 4A; 3A-D |
| p1770 | [pRS413] *MET15* | [[2](#_ENREF_2)] | 3A, B |
| pMP3008 | *[pRS413] LST4p-LST4-V5-HIS_6_* | [[3](#_ENREF_3)] | 4C |
| pMB1353 | *2µ, HIS3, ADH1p-AVT1* | This study | 2E |
| pET-28b | *T7* promoter*, kanR* | Addgene |  |
| pMP3035 | [pET-28b] His_6_-Lst4^loop^ | This study | 4G; 4I |
| pAH051 | *CEN, ARS, URA3, SCH9p-SCH9^T723D,T726D,T737E,T758E,T765E^-HA* | [[4](#_ENREF_4)] | 3A-D |
| pBK549 | CEN, URA3, carries MiniDs in *ADE2* and hyperactive Ac transposase under *GAL1* promoter | [[5](#_ENREF_5)] | 2A-D |

**References**

**1**. Brachmann CB, Davies A, Cost GJ, Caputo E, Li J, Hieter P, et al. Designer deletion strains derived from *Saccharomyces cerevisiae* S288C: a useful set of strains and plasmids for PCR-mediated gene disruption and other applications. Yeast. 1998; 14: 115-132. <https://doi.org/10.1002/(SICI)1097-0061(19980130)14:2><115::AID-YEA204>3.0.CO;2-2 PMID: 9483801

**2**. Hatakeyama R, De Virgilio C. TORC1 specifically inhibits microautophagy through ESCRT-0. Curr Genet. 2019; 65: 1243-1249. <https://doi.org/10.1007/s00294-019-00982-y> PMID: 31041524

**3**. Péli-Gulli MP, Raucci S, Hu Z, Dengjel J, De Virgilio C. Feedback inhibition of the Rag GTPase GAP complex Lst4-Lst7 safeguards TORC1 from hyperactivation by amino acid signals. Cell Rep. 2017; 20: 281-288. <https://doi.org/10.1016/j.celrep.2017.06.058> PMID: 28700931

**4**. Wanke V, Cameroni E, Uotila A, Piccolis M, Urban J, Loewith R, et al. Caffeine extends yeast lifespan by targeting TORC1. Mol Microbiol. 2008; 69: 277-285. <https://doi.org/10.1111/j.1365-2958.2008.06292.x> PMID: 18513215

**5**. Michel AH, van Schie S, Mosbach A, Scalliet G, Kornmann B. Exploiting homologous recombination increases SATAY efficiency for loss- and gain-of-function screening. 2020. <https://doi.org/10.1101/866483>
