# Supplementary material for: Indole-3-acetic acid is a physiological inhibitor of TORC1 in yeast
Source: PLoS Genet. 2021 Mar 9;17(3):e1009414. doi: 10.1371/journal.pgen.1009414 (PMC7978357; doi:10.1371/journal.pgen.1009414)
Supplement: S3 Table — (DOCX) [file pgen.1009414.s003.docx]

**S3 Table. Primers used in the SATAY screening.**

| **Purpose** | **Primer sequence** | **Name** |
| --- | --- | --- |
| Amplify and barcode Regrowth NlaIII sub library | CAA GCA GAA GAC GGC ATA CGA GAT TCG CCT TAA CGA AAA CGA ACG GGA TAA A | P7_indexed_N701 (*) |
| Amplify and barcode Regrowth DpnII sub library | CAA GCA GAA GAC GGC ATA CGA GAT TTC TGC CTA CGA AAA CGA ACG GGA TAA A | P7_indexed_N703 (*) |
| Amplify and barcode No IAA NlaIII sub library | caa gca gaa gac ggc ata cga gat GTA GAG AGa cga aaa cga acg gga taa a | P7_indexed_N707 (*) |
| Amplify and barcode NO IAA DpnII sub library | caa gca gaa gac ggc ata cga gat TGC CTC TTa cga aaa cga acg gga taa a | P7_indexed_N711 (*) |
| Amplify and barcode 1mM IAA NlaIII sub library | caa gca gaa gac ggc ata cga gat CCT GAG ATa cga aaa cga acg gga taa a | P7_indexed_N715 (*) |
| Amplify and barcode 1mM IAA DpnII sub library | caa gca gaa gac ggc ata cga gat TAG CGA GTa cga aaa cga acg gga taa a | P7_indexed_N716 (*) |
| Amplify and barcode 1.5mM IAA NlaIII sub library | caa gca gaa gac ggc ata cga gat GCA GCG TAa cga aaa cga acg gga taa a | P7_indexed_N721 (*) |
| Amplify and barcode 1.5mM IAA DpnII sub library | caa gca gaa gac ggc ata cga gat CGC TCA GTa cga aaa cga acg gga taa a | P7_indexed_N724 (*) |
| Amplify all libraries | AATGATACGGCGACCACCGAGATCTACtccgtcccgcaagttaaata | P5_MiniDs (*) |
| Sequencing of Transposon-genome junction | tttaccgaccgttaccgaccgttttcatcccta | 714 |
| Sequencing of the indexes | GGT TTT CGA TTA CCG TAT TTA TCC CGT TCG TTT TCG T | Custom_index1 |

(*) Oligonucleotide sequences © 2016 Illumina, Inc. All rights reserved. Derivative works created by Illumina customers are authorized for use with Illumina instruments and products only. All other uses are strictly prohibited.
