# Supplementary material for: Indole-3-acetic acid is a physiological inhibitor of TORC1 in yeast
Source: PLoS Genet. 2021 Mar 9;17(3):e1009414. doi: 10.1371/journal.pgen.1009414 (PMC7978357; doi:10.1371/journal.pgen.1009414)
Supplement: S4 Table — (DOCX) [file pgen.1009414.s004.docx]

**S4 Table. Libraries characteristics.**

| **Library** | **# of clones** | **# of generations during** | | | **# of transposons mapped** | **# of reads mapped** | **Mean reads per transposon** | **Median reads per transposon** |
| --- | --- | --- | --- | --- | --- | --- | --- | --- |
|  |  | **Regrowth/ pre-IAA treatment** | **first**  **IAA treatment** | **second IAA treatment** |  |  |  |  |
| No IAA | 3E+07 | 12.7/2.5 | 2.5 | 5.46 | 559039 | 45589230 | 89.82 | 12 |
| 1mM  IAA | 3E+07 | 12.7/2.5 | 2.5 | 4.17 | 357008 | 34207525 | 102.65 | 8 |
